# Perspectives of VA healthcare from rural women veterans not enrolled in or using VA healthcare

**DOI:** 10.1371/journal.pone.0289885

**Published:** 2023-08-14

**Authors:** Carly M. Rohs, Karen R. Albright, Lindsey L. Monteith, Amber D. Lane, Kelty B. Fehling

**Affiliations:** 1 Seattle-Denver Center for Innovation (COIN), Rocky Mountain Regional VA Medical Center, Aurora, Colorado, United States of America; 2 VA Rocky Mountain Mental Illness, Research, Education and Clinical Center (MIRECC) for Suicide Prevention, Aurora, Colorado, United States of America; 3 Division of General Internal Medicine, Department of Medicine, University of Colorado Anschutz Medical Campus, Aurora, Colorado, United States of America; 4 Department of Physical Medicine and Rehabilitation, University of Colorado Anschutz Medical Campus, Aurora, Colorado, United States of America; The University of Melbourne Melbourne Dental School, AUSTRALIA

## Abstract

**Purpose:**

Women Veterans have unique healthcare needs and often experience comorbid health conditions. Despite this, many women Veterans are not enrolled in the Veterans Health Administration (VHA) and do not use VHA services. Underutilization of VHA services may be particularly prevalent among rural women Veterans, who may experience unique barriers to using VHA care. Nonetheless, knowledge of rural women Veterans and their experiences remains limited. We sought to understand rural women Veterans’ perceptions and needs related to VHA healthcare, including barriers to enrolling in and using VHA services, and perspectives on how to communicate with rural women Veterans about VHA services.

**Methods:**

Rural women Veterans were recruited through community engagement with established partners and a mass mailing to rural women Veterans not enrolled in or using VHA healthcare. Ten virtual focus groups were conducted with a total of twenty-nine rural women Veterans (27 not enrolled in VHA care and 2 who had not used VHA care in the past 5 years) in 2021. A thematic inductive analytic approach was used to analyze focus group transcripts.

**Findings:**

Primary themes regarding rural women Veterans’ perceptions of barriers to enrollment and use of VHA healthcare included: (1) poor communication about eligibility and the process of enrollment; (2) belief that VHA does not offer sufficient women’s healthcare services; and (3) inconvenience of accessing VHA facilities.

**Conclusion:**

Although VHA has substantially expanded healthcare services for women Veterans, awareness of such services and the nuances of eligibility and enrollment remains an impediment to enrolling in and using VHA healthcare among rural women Veterans. Recommended strategies include targeted communication with rural women Veterans not enrolled in VHA care to increase their awareness of the enrollment process, eligibility, and expansion of women’s healthcare services. Creative strategies to address access and transportation barriers in rural locations are also needed.

## Introduction

During their military service, many women experience stressful and traumatic experiences, such as military sexual trauma, combat, separation from family and friends, and gender bias [1–3]. These experiences are associated with elevated risk for health concerns, such as chronic pain, depression, substance use disorders, and suicidal ideation [4–8]. Further, studies have noted high rates of comorbid health conditions among women Veterans [9]. As such, it is essential that women Veterans have access to the comprehensive healthcare services provided by the Veterans Health Administration (VHA), which has made concerted efforts to address women Veterans’ specific healthcare needs through women’s health clinics and Patient Aligned Care Teams that work closely with Women Veterans Program Managers (WVPM) in the provision of reproductive healthcare and maternity care coordination [10–12]. The VHA has committed to having a WVPM located in every regional office, whose role is to advise, advocate for, and coordinate all services the women Veteran may need [13].

Despite the expansion of women’s health services within VHA, many women Veterans do not use VHA services [14]. Reasons for this are multi-faceted, including limited knowledge and inaccurate beliefs regarding eligibility for services [15] and their perspectives of their Veteran identity [16]. Accordingly, the VHA Office of Women’s Health (OWH) developed the Women’s Health Transition Training (WHTT) [17] pilot program in 2018 at five Air Force bases to inform women service members about their eligibility for VHA care and provide instructions on how to enroll to receive VHA care. The WHTT also seeks to increase women Veterans’ awareness of VHA healthcare services for women (e.g., reproductive healthcare, maternity care, cancer screenings, military sexual trauma counseling) and change the misperception that VHA is only for men. In June 2019, WHTT became an official VHA program and expanded to the Army, Navy, and Marine Corps [18].

The WHTT has primarily been implemented with women transitioning out of military service. Yet specific groups of women Veterans, such as those residing in rural areas, may experience particularly salient barriers to using VHA services. Barriers for rural Veterans include the need to travel longer distances to receive care [19], fewer physicians, hospitals and health resources in rural areas, and limited broadband internet for telehealth [20]. These challenges can exacerbate health conditions [21, 22]. Thus, initiatives to ensure rural women Veterans’ access to VHA care [22, 23] are essential.

Accordingly, programs that inform women Veterans about VHA service eligibility and comprehensive women’s healthcare services may be particularly important for those living in rural areas. Nonetheless, knowledge of how to tailor such interventions (e.g., WHTT) for rural women Veterans not enrolled in VHA care is lacking. Additionally, although many studies have examined women Veterans’ perceptions of VHA care, there is less knowledge of women *not* enrolled in VHA care [16, 24, 25]. Moreover, despite increased focus on healthcare access and services for women Veterans and rural Veterans, there is limited knowledge of rural women Veterans’ healthcare experiences [22, 23, 26, 27]. Understanding rural women Veterans’ experiences and barriers to enrolling in VHA care is essential to tailoring interventions such as WHTT and successfully engaging more rural women Veterans in VHA healthcare to address their health needs.

In the current manuscript, we describe a VHA quality improvement focus group project conducted with rural women Veterans not enrolled in, or recently using, VHA care. Specifically, we sought to understand rural women Veterans’ perceptions and needs regarding VHA healthcare, including barriers to enrolling in and using VHA care, and their perspectives on how to communicate with rural women Veterans about VHA services.

## Materials and methods

This project was a quality improvement project that was acknowledged as such by the local Department of Veteran Affairs (VA) Research and Development Committee, making it exempt by the local IRB.

### Eligibility

We aimed to conduct focus groups with rural women Veterans who had never enrolled in VHA care or who had not utilized VHA care in the past five years. Following insights from recent work [28] on rural identification, we permitted self-reporting of rural residence, rather than relying upon zip codes in the rural-urban commuting area (RUCA) dataset, to determine geographically rural designations; many women described their residence as rural even if not classified as such by their designated RUCA code [29]. Women had to be at least 18 years of age to participate.

### Recruitment

Recruitment efforts occurred both nationally and in specific locations where the team had established relationships, such as in Colorado, Washington, and Wisconsin. For national recruitment, we utilized Fiscal Year 2019 data from the United States Veterans Eligibility Trends and Statistics (USVETS), an integrated dataset of Veteran demographic data [30], to identify 2,500 rural women Veterans who were not currently enrolled in VHA care, stratified by region. In June and July 2021, we mailed letters and a flyer to these women Veterans to inform them of the focus group opportunity. We also disseminated a flyer to rural Veterans, rural community healthcare providers, and Veteran Service Officers (VSOs) through our team’s community partnerships, Veteran-specific social media pages, and Veteran newsletters. Lastly, we attended women-specific monthly meetings of a Veterans Organization and staffed a booth at a women Veterans focused conference.

### Screening

**Fig 1:**
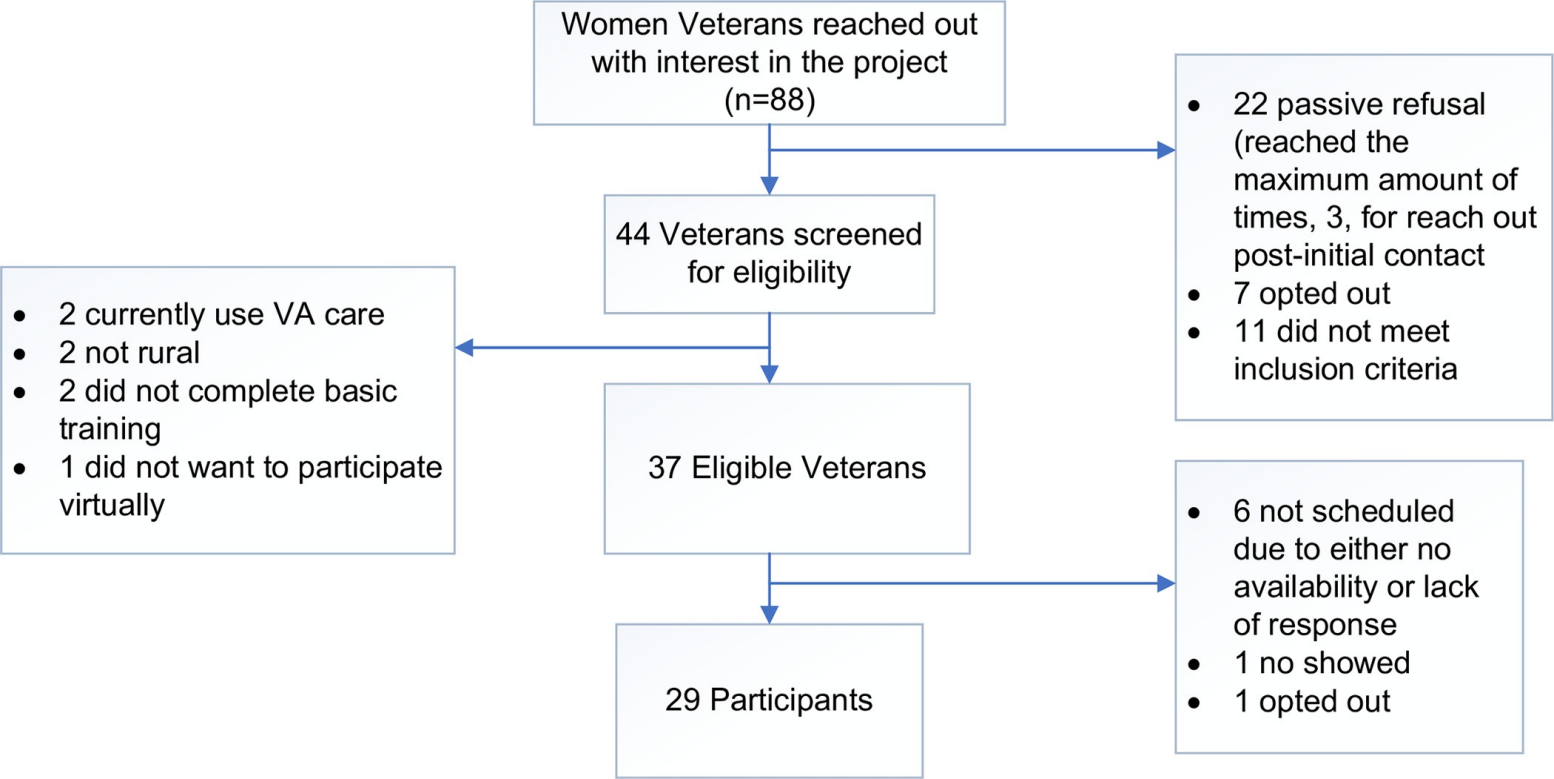
Recruitment consort diagram.

Eighty-eight women Veterans contacted our team to express interest in participating in a focus group. Of those, 42.0% (n = 37) were eligible to participate upon telephone screening, during which a staff member obtained information from them regarding their demographics, military service, and enrollment and use of VHA care. 78.4% (n = 29) of those who were eligible during screening subsequently participated in a focus group. Common reasons for ineligibility included being unable to join the focus group virtually and no longer living in a rural location (Fig 1). Of focus group participants, the majority (n = 23; 79.3%) were recruited through USVETS mailings, while a smaller proportion were recruited through community engagement (n = 6; 20.7%).

### Focus groups

In June and July 2021, we conducted 10 focus groups. Focus groups were selected because of their utility in investigating complex behavior and motivations about health behaviors [31], even when consensus among group members may be low [32]. Given COVID-19 constraints, focus groups were conducted virtually. Discussions were facilitated using a semi-structured guide (S1 File) by one of two facilitators. One facilitator was an established qualitative researcher; the other facilitator was a VA psychologist with content expertise regarding both women Veterans and rural Veterans, as well as experience qualitatively interviewing women Veterans. Information about the facilitators was provided to focus group participants. An additional team member took notes and managed technology. Focus groups were recorded for transcription and analysis, and informed verbal consent was obtained from focus group participants for taking part in the focus groups and recording of them. Recommended best practices for virtual data collection were followed [33, 34], including keeping focus groups small (2–4 Veterans per group) to facilitate deeper discussion. Each group lasted approximately 90 minutes. Veterans were emailed VHA resources (including information regarding resources for women Veterans) following each group.

### Analysis

Consistent with established qualitative methodology, analysis was conducted as a continuous process beginning with initial focus groups and continuing throughout and beyond the data generation period. Analysis occurred in an iterative and team-based process involving established qualitative content methods and reflexive team analysis [35, 36], led by an experienced qualitative methodologist (one of the focus group facilitators). Each transcript was read multiple times to achieve immersion prior to code development. The qualitative methodologist then derived an initial code list deductively, based on domains included in the focus group guide, and inductively, based on additional domains emergent in transcripts. Following discussion and refinement by the project team, the final coding schema was applied to each transcript by the methodologist and analyzed together for patterns using the qualitative data software package ATLAS.ti [37, 38]. Throughout this process, team members met regularly to discuss emergent codes and themes and to assess preliminary results [39]. Thematic saturation was determined when repeated analysis yielded no new themes. Qualitative findings are reported according to the Consolidated Criteria for Reporting Qualitative Research (COREQ) 32-item qualitative standards checklist (S2 File) [40].

## Results

### Characterizing the sample: Demographics, military service, and VHA use

Participant characteristics are presented in Table 1. Participants tended to be older, and the majority were currently married. Racial and ethnic diversity was limited, with the majority identifying their race as White and none reporting Hispanic ethnicity. The majority were currently employed or retired. Branch of military service varied, with Army and Air Force most common. Most participants had not deployed. Time in service varied broadly. Most participants reported being honorably discharged. In terms of VHA enrollment and use, most participants have never enrolled in (or used) VHA care, while a small minority had previously enrolled in VHA care, though had not used any VHA services in the past five years. Focus group participants resided in the following states: Alaska (1), Washington (5), Oregon (1), Idaho (2), Montana (2) Wyoming (2), Utah (1), Colorado (2), Arizona (1), Nebraska (1), Iowa (1), Minnesota (2), Wisconsin (1), Illinois (2), Mississippi (1), Massachusetts (1), New Hampshire (1) and Maine (2).

**Table 1:** Focus group participant characteristics (n = 29).

Characteristic	M	SD	n (%)
**Age** (years)	60	± 10	-
**Military Service** (years)	7	± 6	-
**Marital Status**	-	-	
Married			23 (79%)
Single and never married			4 (14%)
Divorced			0 (0%)
Widowed			2 (7%)
**Hispanic/Latina**	-	-	
Yes			0 (0%)
No			29 (100%)
**Race**	-	-	
American Indian or Alaska Native			1 (3%)
Black or African American			2 (7%)
White			26 (90%)
**Employment**	-	-	
Employed full-time			12 (41%)
Employed part-time			4 (14%)
Not employed, not currently seeking assistance			2 (7%)
Retired from the workforce			11 (38%)
**Military Branch**	-	-	
Air Force			9 (31%)
Army or Army National			16 (55%)
Guard			1 (3%)
Marine Corps			3 (11%)
Navy			
**Ever Deployed**	-	-	
Yes			8 (28%)
No			21 (72%)
**Discharge Status**	-	-	
Honorable			27 (93%)
General (Medical)			1 (3%)
Other than Honorable			1 (3%)
**VHA Enrollment and Use**	-	-	
Never enrolled, no use			27 (93%)
No use in the last five years			2 (7%)

### Overall focus group findings

Three primary themes emerged. First, participants reported poor communication about their eligibility for VHA services and the enrollment process. Second, they expressed the belief that VHA did not offer sufficient women’s healthcare services. Finally, they perceived the inconvenience of accessing VHA facilities as a barrier to using VHA healthcare.

#### Poor communication about eligibility and enrollment

An overwhelming majority of participants indicated a lack of communication and/or miscommunication regarding their eligibility for VHA healthcare and the process of determining if they were eligible to obtain VHA services. Indeed, not a single participant described perceiving that there had been clear communication about eligibility. Instead, confusion about this was typically cited as the primary barrier to enrollment in VHA care. Veterans described their strong perception that, in their own experience and the experiences of other women Veterans whom they had interacted with, information about what VHA services they qualified for and how to access services (i.e., process, location) was not disseminated effectively across VA or military systems. This resulted in the perception of opaque and/or inequitable eligibility.

*I haven’t filled out the [VA healthcare] paperwork just cause, you know, it’s paperwork…and then you get a rejection or no, you’re not eligible, and you’ve put five hours into filling out… 50 pages of whatever, and it just seems like bureaucracy again. It’s the whole process by which you… why not just make it automatic, why not just tell everybody, hey, you’re, you’re in the VA system. Just go and show up. Why do you have to fill out all this paperwork to prove that you’re a person that got out of the military?… In this day and age with computers and the fact that you [project team] found us, why isn’t the VA finding us?*

To improve understanding of VHA eligibility and streamline the process for determining and/or obtaining services, participants emphasized the need for widespread marketing and consistent messaging as well as, in some cases, more individualized communication and outreach to women Veterans to inform them of services available, eligibility nuances, and VHA points of contact for interfacing with the system. Participants suggested immediately preceding military discharge as one critical time to effectively communicate such information, since transitioning service members are a receptive population and have not yet disbursed, taken new employment, and/or obtained other health insurance. Participants noted that it is precisely then that women most need to understand what they will qualify for after discharge and what exactly they must do to access care.

*Facilitator*: *So*, *it sounds like there’s a theme that’s emerging here*, *that there’s some miscommunication or lack of communication about eligibility*, *about process*, *just about all the details*.

*Participant 1*: *Yeah*. *That actually could be helpful when you get out [of the military at discharge] …if somebody wants benefits or understanding how they could get them*.

*Participant 2*: *It would’ve been nice…when I got discharged if somebody would’ve said*, *hey… don’t forget*, *you’re eligible for this*. *Here’s how you look into it… give you some little heads-up*, *but I got nothing*.

Because of the clarity needed for this information, Veterans also suggested that better training about eligibility and enrollment should be required for branch officers tasked with discharge, since those points of contact should be able to answer basic questions and provide initial guidance about next steps for enrolling in VHA healthcare.

*Participant 1*: *When I separated*, *they didn’t say what*, *what the benefits were…what your eligibility was*.

*Participant 2*: *I’m not sure if it was more the VA or if it was more of the military itself*, *the Air Force itself*. *Because*, *I mean*, *they’re the ones that gave me my discharge papers… And they’re the ones that gave me the impression that I was not eligible for anything unless it happened while I was in the military*.

*Participant 3*: *I was gonna say the exact same thing*. *I’m not sure they really knew*. *They should know so they can tell people*.

Military separation was not the only time noted, however, as important for obtaining information regarding VHA eligibility and enrollment. Women also expressed a desire to receive information about their eligibility for VHA services around key transitions in their lives, such as retirement from subsequent (non-military) employment, as this often represented a period of transition with substantial impacts to their healthcare coverage.

*Participant*: *Yeah*, *remind them*, *and tell them that there is a way to find out more information about it if they’re interested*. *I know maybe had I gotten that ten years ago or something*, *I might have… checked it out more*, *you know*, *because health does change when you get older… When you’re in your 30s and 40s*, *you may not think about it so much if you’re… out by then*, *but… when you hit your 70s*, *things start escalating and changing*, *so I think that routinely*, *letters should be sent out so that Veterans do know what is available*.

Women also emphasized the need for the VA to reach out to women Veterans through multiple modalities, with clear information about eligibility and the process of determining it. Various methods for sharing information about VHA services offered, eligibility, and enrollment processes were suggested. Most emphasized was the need to improve and simplify the VA website so the process of determining VHA eligibility is clear, including providing contact numbers or email addresses of people whom Veterans can communicate with concerning personal questions.

*Participant 1*: *I don’t think you can go to an online thing and find out automatically*, *quickly*, *if you are eligible for services*. *That would’ve been awesome*, *and I think that that would be true for a lot of people if there was a quick way of finding out that you’re eligible*, *like just on the website*, *then I think more people would access it*.

*Participant 2*: *That’s what we need*. *End the uncertainty*, *just making it quick*, *easy*. *You go here*, *and then you get an answer right away*. *Or even just a name and number*, *a real person we can direct contact*.

Other suggested methods included direct mailings to Veterans, direct outreach to Veteran Service Organizations and/or local care facilities and other rural community resources, and advertising in communities, Veteran newsletters, and television.

*Participant 1*: *I think there’s two times [when it would be helpful to receive information about VA healthcare]*. *Number one is when someone is discharged*, *for them to get a whole packet giving them all the information about the VA and their options*. *And then… maybe at some point*, *there should be a general letter sent out to Veterans… maybe every ten years or whatever*, *they send out a big blanket folder and things to let people know about the services that they could get through the VA and tell them that please apply*, *and you’ll get further information [about eligibility]*.

#### Perception of lack of women-focused care

Many focus group participants reported the perception that VHA does not provide healthcare oriented to women. Some cited this as disincentive to enrolling in or using VHA healthcare.

*Participant 1*: *They never show you any women Veterans in those news stories [about VA healthcare]*. *They’re always men*. *So women Veterans*, *they’re a very well-kept secret because we know nothing about them*.

*Participant 2*: *I agree 100%*, *100%*.

*Participant 3*: *I agree as well*. *And out of all the women Veterans I know*, *I don’t know any of them that go to the VA*. *And I’ve been in four or five different states with my careers*, *and it’s all these Veterans*, *they don’t go to the VA*, *and so even the ones that have retired don’t use the VA*.

Some participants explicitly associated VHA with Veteran men, particularly those of older generations.

*Facilitator*: *What are your impressions or your thoughts when you think about VA health care*?

*Participant 1*: *Old white men*.

*Facilitator*: *Old white men*. *OK*. *You mean as the doctor*, *or as the provider*, *or*?

*Participant 1*: *No*, *as the patients*, *and you know*, *when I get around a bunch of Veterans*, *like VFW Veterans or Legion Vets*, *it gives me flashbacks to when I was in the military and had to put up with sexism and stuff like that*, *so it doesn’t really jive for me*.

*Participant 2*: *I think*, *even when I was active duty and working*, *the thought of having to enter the VA system was honestly a bit sketchy to me and scary because it was*, *you know*, *a bunch of old men dying in hospitals is what it felt like to me*…

Some participants cited concerns about the quality of women’s healthcare services within VHA, as well as regarding insensitivity to women’s privacy needs.

*Participant 1*: *I wouldn’t go to the VA if I had breast cancer… I wouldn’t trust the VA with that*. *I would probably go more with a specialist or somebody who is more in line with that type of care*… *If I had actually known that there was somebody right down the street that I could go see*, *I would probably only see the VA mostly for check-ups*.

*Facilitator*: *And is that because*, *is it a specialization issue*, *like if there was a specialist at the VA in that*, *that would be fine*? *Or is it a concern about possible quality of care*?

*Participant 1*: *If I found a specialist that leaned that direction*, *I would probably feel a little more comfort for it*, *but I would venture to say it’s mostly on the horror stories of quality of care and past experience that I would most likely go a different direction*.

*Participant 2*: *The year I was [in Veteran Service Organization*, *we had town hall meetings*, *and we were told by VA reps that the only thing that was available for female Veterans for their yearly breast and gyn exams were telehealth*, *and I said you expect me to go out and tell my female Veterans that they’re going to have to be on a television screen for their breast or gyn exam*, *and the answer was yes… so I think they’re insensitive to it as well*.

For the aforementioned reasons, many participants expressed strong interest in women’s specialty clinics. Most focus group participants were not aware that women’s specialty clinics exist within VHA facilities. Some also indicated support for having more female physicians in VHA.

*Participant 1*: *I think that [getting care at a VA women’s clinic] would be lots more comfortable for all of us*, *well*, *it certainly would be for me*, *but*, *you know*, *with the current system*, *I don’t think that’s out there anywhere*, *is it*?

*Participant 2*: *With more women physicians*.

*Participant 3*: *I don’t care if I have a male or a woman physician*, *as long as they’re there to do their job*, *and they do their job well*, *looking after me the way I need to be looked after and not making me feel like a number*. *But a women-only location*, *I think that that would actually be an amazing idea because then they’re specialized in that one field*. *I mean*, *we are completely different than men… I would say that yeah*, *a woman-only clinic would be an amazing idea*.

Participants stressed that, to increase women Veterans’ use of VHA services, any outreach and/or advertising should explicitly present the VHA as a place of care for women, to counteract the association of VHA as a bastion of “old White men.” This would include advertisement of women’s clinics and services available within VHA.

#### Inconvenience of accessing services

A third and final barrier centered on the inconvenience of accessing VHA services. Some participants noted that many VHA facilities were geographically distant from the rural communities in which they lived and thus took significant time to drive to.

*I was told that I had to have my initial evaluation by a civilian provider that was on the [direction] side of [city]*, *which is a four-hour drive from my home*, *and I had an 8*:*00 in the morning appointment*, *and so I asked could I make arrangements to go the evening before and be there*, *and I was told*, *well*, *I could*, *but I’d have to foot the bill*, *there were nothing to help compensate for it*, *and I said well*, *there’s a VA clinic in [a smaller city]*, *which is 65 miles away and then there’s one in [city] which is about 75 miles away*, *and I said I could go to either of those*, *and I could drive that relatively easy and be there by 8*:*00 in the morning*, *and they said no*, *that’s not where you’ll go*. *You have to go to this place on the [direction] side of [city]*, *and I said*, *I’m not going there*, *and they said*, *OK*, *we’re going to put down on your application that you refused the appointment that we gave you*.

Some focus group participants also reported perceived difficulty getting timely appointments as a barrier to using VHA care.

*For me*, *it’s a lack of care*. *I know lots and lots of people who are within the VA*, *but they still can’t get an appointment*. *You had a high blood pressure*, *you have a nosebleed*, *and they say no*, *you’re not authorized to go to the emergency room*. *You’ve got to wait and come to our clinic on Monday when you can ask for an appointment*, *and the appointment is six weeks down the road*. *So*, *for me*, *it’s the inability of availability*.

Further, some participants reported that technological limitations in their rural areas made virtual appointments difficult. For example, some had difficulty accessing broadband internet from home, and expressed the desire for other (e.g., mobile) options for in-person care:

*Participant*: *It’s a two-hour drive to go to a VA center*, *so not that small towns necessarily need a permanent VA structure*, *but they should have that mobile bus…*. *[the public health department] will come out here and do mobile mammograms*, *and the VA should do the same thing*. *They should have a mobile office that hits these small-town areas because it is a burden for somebody to have to drive two hours to go to the doctor*.

*Facilitator*: *If the VA offered any services to women Veterans in rural areas that could be delivered virtually*, *is that something that would be of interest or not*?

*Participant*: *At home*, *I have satellite internet*. *Virtual doctor visits and things like that don’t work well*. *I happen to be in town for this [virtual focus group] right now*, *so that way I can…basically [use] somebody else’s internet for this…*.*because it would use all my data at home*.

Several others indicated that, even if VHA telehealth services were possible, they would not want to use telehealth services, as telehealth was not perceived as appropriate (e.g., OB/GYN) or personal enough (e.g., mental health) for their healthcare needs.

Because of the perceived inconveniences of accessing VHA services, some focus group participants who had other health insurance options preferred to receive care at a closer non-VHA facility. Participants also noted that many Veterans receive healthcare through both private healthcare organizations and VHA, and that better communication and coordination between them is necessary to reduce record scatter (different records in multiple systems), focus resources, and improve patient service and experiences. Similarly, some emphasized the need to build and/or strengthen connections to and partnerships with local community care facilities to improve healthcare access. These participants described a desire for a system in which they could show up to any healthcare facility with a Veteran card and receive healthcare there. However, a few noted concerns about privacy within their rural communities and noted that, as such, they would like to be able to access VHA healthcare, if eligible.

## Discussion

Perceptions about poor communication about eligibility and enrollment, insufficient women’s healthcare services, and inconvenience of accessing VHA facilities were described as barriers to enrolling in and using VHA care among rural women Veterans who participated in our focus groups.

### Improving communication

Our findings suggest that lack of accurate information regarding eligibility for VHA healthcare and enrollment, as well as regarding healthcare services available to women Veterans, precluded rural women Veterans from enrolling in and using VHA services. Additionally, consistent with prior studies of women Veterans who utilized VHA care [41, 42], rural, unenrolled women Veterans believed that VHA does not provide healthcare relevant to women Veterans’ unique needs and that VHA is a place for “old White men,” despite significant VHA expansion of healthcare services for women Veterans to include comprehensive women’s health services, maternity care coordination, and provision of specialized training to VHA Women’s Health providers in women Veterans’ healthcare needs [43–46]. As many women Veterans have experienced their needs as being disregarded or have experienced prior interpersonal or institutional trauma [47–49], these perceptions may be particularly detrimental, as negative perceptions regarding VHA care can result in delayed or forgone healthcare use [50, 51].

Accordingly, it is critical to ensure that accurate information is widely available to rural women Veterans regarding VHA eligibility, the process of VHA enrollment, and women’s health services. Such information should be easily accessible, consistently updated, and disseminated in simple, clear terms through multiple modalities. Updates should be disseminated to rural women Veterans, irrespective of when they served, and to those who interact with rural women Veterans. For example, VSOs, community healthcare providers, and branch officers involved in military separation may be important conduits of such knowledge. Local VHA facilities could also periodically reach out to rural women Veterans through targeted mailings or community events to provide information regarding services available to women Veterans, how to access those services, as well as specific ways in which VHA is responding to women Veterans’ healthcare needs and concerns. For example, VHA has implemented broad initiatives to prevent harassment at VHA facilities and ensure its facilities are welcoming to women Veterans. As rural women Veterans in our sample were generally unaware of these efforts, such information could be more widely disseminated outside of VHA.

Efforts to communicate such information with rural women Veterans should consider the timing of such communications. Although some women Veterans in our sample reported that they did not need to use VHA services in the period following separation from the military, a portion described later needing healthcare services, indicating that they would have used or considered using VHA care at other points in their lives, if they were eligible to use VHA care. However, many were unsure if they were eligible or did not know how to enroll in VHA care, and women were largely unaware that eligibility can change over time or be offered for specific circumstances or types of care (e.g., healthcare for conditions related to military sexual trauma; time-limited mental healthcare). Thus, targeted communications with rural women Veterans not enrolled in or using VHA services could emphasize both the period preceding military separation, as well as other life transitions where healthcare services may be particularly important (e.g., employment-related transitions, such as retirement). Additionally, such communications could clarify exceptions to broader eligibility to inform women Veterans of exceptions to eligibility for VHA care and circumstances in which their eligibility could change.

### Inconvenience of accessing VHA services

Lack of access and transportation, which have been noted to also impede use of healthcare services for rural Veterans in other studies [52–54], were noted barriers among rural women Veterans to using VHA services in the present sample. Thus, it is critical to determine feasible, acceptable, and effective ways to deliver healthcare to rural women Veterans. Telehealth may be a particularly important modality of healthcare among rural women Veterans, particularly considering barriers within this population (e.g., caregiving) which may further compound access-related challenges [55]. As access to reliable broadband internet in rural areas can impede access to telehealth, it may be important to establish hubs in rural communities where Veterans can privately access internet for telehealth. Additionally, it may be particularly important to increase rural women Veterans’ awareness of the VA Digital Divide program, which offers discounts on home internet and phone services or provides a device with internet connectivity for Veterans to engage in telehealth [56]. While VHA offers some such services at community-based outpatient clinics (CBOCs), it may be helpful to also establish these in easily accessible locations which do not require extensive transportation to access. Communication between VHA and community healthcare organizations is critical to ensure service coordination and provision. Finally, in communities that offer transportation for rural Veterans (e.g., carpooling to VHA facilities), it is important to ensure that women Veterans, VSOs, and community providers are all aware of these offerings.

### Limitations

One major limitation to our findings is that some women whom we interviewed reported that they were not eligible for VHA care, yet we were unable to verify this and did not ask women to elaborate on the last time they had applied for VHA care. Eligibility has expanded widely, and thus many of these women may now be eligible. Findings are also limited by the focus on specific geographic areas and the lack of racial and ethnic diversity within our sample. Additional research is needed to understand how findings extend to rural women Veterans of other racial (e.g., Black, Asian American, Pacific Islander, American Indian, Native Alaskan) and ethnic (i.e., Hispanic) backgrounds, and to understand the perceptions and experiences of rural women Veterans in other geographic areas where there may be additional concerns and barriers to enrolling in VHA care (e.g., US Territories).

## Conclusion

Rural women Veteran focus group participants reported several barriers to enrollment and use of VHA care, including poor communication regarding VHA eligibility and enrollment, negative perceptions of VHA services for women, and inconvenient access to VHA facilities. These barriers can be addressed by improving communication about VHA eligibility and enrollment, increasing awareness regarding VHA healthcare services for women Veterans, and addressing barriers related to access and transportation in rural locations. These findings and recommendations can be used to inform modifications to the WHTT intervention, as well as development of improved marketing and dissemination of information to rural, unenrolled women Veterans to ensure they can access VHA healthcare when needed.

## Supporting information

S1 File Focus group interview guide. (PDF)

S2 File Consolidated criteria for reporting qualitative research. (PDF)

## References

[1] Zinzow HM, Grubaugh AL, Monnier J, Suffoletta-Maierle S, Frueh BC. Trauma Among Female Veterans: A Critical Review. Trauma Violence Abuse. 2007 Oct;8(4):384–400. doi: 10.1177/1524838007307295 17846179

[2] Street AE, Vogt D, Dutra L. A new generation of women veterans: stressors faced by women deployed to Iraq and Afghanistan. Clin Psychol Rev. 2009 Dec;29(8):685–94. doi: 10.1016/j.cpr.2009.08.007 19766368

[3] Mattocks KM, Haskell SG, Krebs EE, Justice AC, Yano EM, Brandt C. Women at war: Understanding how women veterans cope with combat and military sexual trauma. Soc Sci Med. 2012 Feb;74(4):537–45. doi: 10.1016/j.socscimed.2011.10.039 22236641

[4] Sumner JA, Lynch KE, Viernes B, Beckham JC, Coronado G, Dennis PA, et al. Military Sexual Trauma and Adverse Mental and Physical Health and Clinical Comorbidity in Women Veterans. Womens Health Issues. 2021 Nov;31(6):586–95. doi: 10.1016/j.whi.2021.07.004 34479786

[5] Cichowski SB, Rogers RG, Clark EA, Murata E, Murata A, Murata G. Military Sexual Trauma in Female Veterans is Associated With Chronic Pain Conditions. Mil Med. 2017 Sep;182(9):e1895–9. doi: 10.7205/MILMED-D-16-00393 28885952

[6] Gilmore AK, Brignone E, Painter JM, Lehavot K, Fargo J, Suo Y, et al. Military Sexual Trauma and Co-occurring Posttraumatic Stress Disorder, Depressive Disorders, and Substance Use Disorders among Returning Afghanistan and Iraq Veterans. Womens Health Issues. 2016 Sep;26(5):546–54. doi: 10.1016/j.whi.2016.07.001 27528358 PMC5026917

[7] Godfrey KM, Mostoufi S, Rodgers C, Backhaus A, Floto E, Pittman J, et al. Associations of military sexual trauma, combat exposure, and number of deployments with physical and mental health indicators in Iraq and Afghanistan veterans. Psychol Serv. 2015 Nov;12(4):366–77. doi: 10.1037/ser0000059 26524278

[8] Monteith LL, Bahraini NH, Matarazzo BB, Gerber HR, Soberay KA, Forster JE. The influence of gender on suicidal ideation following military sexual trauma among Veterans in the Veterans Health Administration. Psychiatry Res. 2016 Oct;244:257–65. doi: 10.1016/j.psychres.2016.07.036 27504921

[9] Creech SK, Pulverman CS, Crawford JN, Holliday R, Monteith LL, Lehavot K, et al. Clinical Complexity in Women Veterans: A Systematic Review of the Recent Evidence on Mental Health and Physical Health Comorbidities. Behav Med. 2021 Jan 2;47(1):69–87. doi: 10.1080/08964289.2019.1644283 31403895

[10] VHA Handbook 1330.03: Maternity Health Care and Coordination 2012. Washington, DC: Department of Veteran Affairs, Veterans Health Administration; 2012.

[11] Zephyrin LC, Katon JG, Hoggatt KJ, Balasubramanian V, Saechao F, Frayne SM, et al. State of Reproductive Health in Women Veterans—VA Reproductive Health Diagnoses and Organization of Care. Women’s Health Services, Veterans Health Administration, Department of Veteran Affairs; 2014 Feb.

[12] Katon JG, Zephyrin L, Meoli A, Hulugalle A, Bosch J, Callegari L, et al. Reproductive health of women Veterans: a systematic review of the literature from 2008 to 2017. In: Seminars in reproductive medicine. Thieme Medical Publishers; 2018. p. 315–22. 10.1055/s-0039-1678750 PMC6613775 31003246

[13] U.S. Department of Veterans Affairs. Women Veterans. Veterans Benefits Administration.

[14] Frayne SM, Phibbs CS, Saechao F, Friedman SA, Shaw JG, Romodan Y, et al. Sourcebook: Women Veterans in the Veterans Health Administration, Volume 4, Longitudinal Trends in Sociodemographics, Utilization, Health Profile, and Geographic Distribution. Women’s Health Eval Initiat Women’s Health Serv Veterans Health Adm Dep Veterans Aff. 2018;144.

[15] Evans EA, Tennenbaum DL, Washington DL, Hamilton AB. Why Women Veterans Do Not Use VA-Provided Health and Social Services: Implications for Health Care Design and Delivery. J Humanist Psychol. 2019 May 17;002216781984732.

[16] Di Leone BAL, Wang JM, Kressin N, Vogt D. Women’s veteran identity and utilization of VA health services. Psychol Serv. 2016 Feb;13(1):60–8. doi: 10.1037/ser0000021 25729892

[17] VA Women’s Health Transition Training, Center for Women Veterans (CWV) [Internet]. U.S. Department of Veteran Affairs. Available from: https://www.va.gov/womenvet/whtt/index.asp

[18] Taylor VH. DoD partners with VA, implements Women’s Health Transition Training Program [Internet]. Air Force Material Command. 2019 [cited 2023 May 23]. Available from: https://www.afmc.af.mil/News/Article-Display/Article/1908167/dod-partners-with-va-implements-womens-health-transition-training-program/#:~:text=In%20July%202018%2C%20the%20VA%20Women%E2%80%99s%20Health%20Transition,ownership%20to%20the%20Veterans%20Benefits%20Administration%20in%202021.

[19] Weeks WB, Kazis LE, Shen Y, Cong Z, Ren XS, Miller D, et al. Differences in Health-Related Quality of Life in Rural and Urban Veterans. Am J Public Health. 2004 Oct;94(10):1762–7. doi: 10.2105/ajph.94.10.1762 15451747 PMC1448531

[20] Rural Veteran Health Care Challenges [Internet]. Office of Rural Health. 2021 [cited 2022 Mar 15]. Available from: https://www.ruralhealth.va.gov/aboutus/ruralvets.asp#vet

[21] Cordasco KM, Mengeling MA, Yano EM, Washington DL. Health and Health Care Access of Rural Women Veterans: Findings From the National Survey of Women Veterans: Health and Health Care of Rural Women Veterans. J Rural Health. 2016 Sep;32(4):397–406. 27466970 10.1111/jrh.12197

[22] Brooks E, Dailey N, Bair B, Shore J. Rural Women Veterans Demographic Report: Defining VA Users’ Health and Health Care Access in Rural Areas: Rural Women Veterans. J Rural Health. 2014 Apr;30(2):146–52. 24689540 10.1111/jrh.12037

[23] Brooks E, Dailey NK, Bair BD, Shore JH. Listening to the Patient: Women Veterans’ Insights About Health Care Needs, Access, and Quality in Rural Areas. Mil Med. 2016 Sep;181(9):976–81. doi: 10.7205/MILMED-D-15-00367 27612340

[24] MarshallV, StryczekKC, HaverhalsL, YoungJ, AuDH, HoPM, et al. The Focus They Deserve: Improving Women Veterans’ Health Care Access. Womens Health Issues Off Publ Jacobs Inst Womens Health. 2021;31(4):399–407. doi: 10.1016/j.whi.2020.12.011 33582001

[25] Kinney RL, Haskell S, Relyea MR, DeRycke EC, Walker L, Bastian LA, et al. Coordinating women’s preventive health care for rural veterans. J Rural Health. 2022 Jun;38(3):630–8. doi: 10.1111/jrh.12609 34310743

[26] Ingelse K, Messecar D. Rural Women Veterans’ Use and Perception of Mental Health Services. Arch Psychiatr Nurs. 2016 Apr;30(2):244–8. doi: 10.1016/j.apnu.2015.11.008 26992878

[27] Murray-Swank NA, Dausch BM, Ehrnstrom C. The mental health status and barriers to seeking care in rural women veterans. J Rural Ment Health. 2018 Apr;42(2):102–15.

[28] Onega T, Weiss JE, Alford‐Teaster J, Goodrich M, Eliassen MS, Kim SJ. Concordance of Rural‐Urban Self‐identity and ZIP Code‐Derived Rural‐Urban Commuting Area (RUCA) Designation. J Rural Health. 2020 Mar;36(2):274–80. doi: 10.1111/jrh.12364 30913340 PMC6763368

[29] Rural-Urban Commuting Area Codes [Internet]. USCA Economic Research Service, U.S. Department of Agriculture. 2020 [cited 2021 Dec 15]. Available from: https://www.ruralhealth.va.gov/aboutus/ruralvets.asp#vet

[30] USVETS [Internet]. Office of Enterprise Integration, Office of Data Governance and Analytics. Available from: https://www.va.gov/oei/

[31] Cote-Arsenault D, Morrison-Beedy D. Practical Advice for Planning and Conducting Focus Groups: Nurs Res. 1999 Sep;48(5):280–3. 10.1097/00006199-199909000-00009 10494913

[32] Morgan DL, Krueger RA. When to Use Focus Groups and Why. In: Successful Focus Groups: Advancing the State of the Art [Internet]. 2455 Teller Road, Thousand Oaks California 91320 United States: SAGE Publications, Inc.; 1993 [cited 2021 Sep 15]. p. 3–19. Available from: http://methods.sagepub.com/book/successful-focus-groups/n1.xml

[33] Santhosh L, Rojas JC, Lyons PG. Zooming into Focus Groups: Strategies for Qualitative Research in the Era of Social Distancing. Sch. 2021 Jun;2(2):176–84. doi: 10.34197/ats-scholar.2020-0127PS 34409412 PMC8357072

[34] Richard B, Sivo SA, Ford RC, Murphy J, Boote DN, Witta E, et al. A Guide to Conducting Online Focus Groups via Reddit. Int J Qual Methods. 2021 Jan 1;20:160940692110122.

[35] Graneheim UH, Lundman B. Qualitative content analysis in nursing research: concepts, procedures and measures to achieve trustworthiness. Nurse Educ Today. 2004 Feb;24(2):105–12. doi: 10.1016/j.nedt.2003.10.001 14769454

[36] Hsieh HF, Shannon SE. Three Approaches to Qualitative Content Analysis. Qual Health Res. 2005 Nov;15(9):1277–88. doi: 10.1177/1049732305276687 16204405

[37] Bradley EH, Curry LA, Devers KJ. Qualitative Data Analysis for Health Services Research: Developing Taxonomy, Themes, and Theory. Health Serv Res. 2007 Aug;42(4):1758–72. doi: 10.1111/j.1475-6773.2006.00684.x 17286625 PMC1955280

[38] Carey M, Morse J. The group effect in focus groups: planning, implementing, and interpreting focus group research. In: Criticial Issues in Qualitative Research Methods. Thousand Oaks, Calif: Sage Publications; 1994. p. 225–41.

[39] Charmaz K. Constructing Grounded Theory: A practical guide through qualitative analysis Kathy Charmaz Constructing Grounded Theory: A practical guide through qualitative analysis Sage 224 £19.99 0761973532 0761973532. Nurse Res. 2006 Jul;13(4):84–84.27702218

[40] Tong A, Sainsbury P, Craig J. Consolidated criteria for reporting qualitative research (COREQ): a 32-item checklist for interviews and focus groups. Int J Qual Health Care. 2007 Sep 16;19(6):349–57. doi: 10.1093/intqhc/mzm042 17872937

[41] Kehle-Forbes SM, Harwood EM, Spoont MR, Sayer NA, Gerould H, Murdoch M. Experiences with VHA care: a qualitative study of U.S. women veterans with self-reported trauma histories. BMC Womens Health. 2017 Dec;17(1):38. doi: 10.1186/s12905-017-0395-x 28558740 PMC5450063

[42] Washington DL, Kleimann S, Michelini AN, Kleimann KM, Canning M. Women Veterans’ Perceptions and Decision-Making about Veterans Affairs Health Care. Mil Med. 2007 Aug;172(8):812–7. doi: 10.7205/milmed.172.8.812 17803071

[43] deKleijn M, Lagro-Janssen ALM, Canelo I, Yano EM. Creating a Roadmap for Delivering Gender-sensitive Comprehensive Care for Women Veterans: Results of a National Expert Panel. Med Care. 2015 Apr;53(Supplement 4Suppl 1):S156–64. doi: 10.1097/MLR.0000000000000307 25767971 PMC4379113

[44] Than C, Chuang E, Washington DL, Needleman J, Canelo I, Meredith LS, et al. Understanding Gender Sensitivity of the Health Care Workforce at the Veterans Health Administration. Womens Health Issues Off Publ Jacobs Inst Womens Health. 2020 Apr;30(2):120–7. 10.1016/j.whi.2020.01.001 PMC8025774 32094056

[45] Women Veterans Health Care; Provider and Nurse Training on Women’s Health Continues to be on the Move in Rural Areas [Internet]. Women’s Health, U.S. Department of Veterans Affairs. 202AD [cited 2022 Mar 15]. Available from: https://www.womenshealth.va.gov/WOMENSHEALTH/LatestInformation/news.asp

[46] Newins AR, Wilson SM, Hopkins TA, Straits-Troster K, Kudler H, Calhoun PS. Barriers to the use of Veterans Affairs health care services among female veterans who served in Iraq and Afghanistan. Psychol Serv. 2019 Aug;16(3):484–90. doi: 10.1037/ser0000230 29419309

[47] Huynh-Hohnbaum ALT, Damron-Rodriguez J, Washington DL, Villa V, Harada N. Exploring the Diversity of Women Veterans’ Identity to Improve the Delivery of Veterans’ Health Services. Affilia. 2003 May;18(2):165–76.

[48] Monteith LL, Holliday R, Schneider AL, Miller CN, Bahraini NH, Forster JE. Institutional betrayal and help-seeking among women survivors of military sexual trauma. Psychol Trauma Theory Res Pract Policy. 2021 Oct;13(7):814–23. doi: 10.1037/tra0001027 33764096

[49] Klap R, Darling JE, Hamilton AB, Rose DE, Dyer K, Canelo I, et al. Prevalence of Stranger Harassment of Women Veterans at Veterans Affairs Medical Centers and Impacts on Delayed and Missed Care. Womens Health Issues. 2019 Mar;29(2):107–15. doi: 10.1016/j.whi.2018.12.002 30686577

[50] Wagner C, Dichter ME, Mattocks K. Women Veterans’ Pathways to and Perspectives on Veterans Affairs Health Care. Womens Health Issues. 2015 Nov;25(6):658–65. doi: 10.1016/j.whi.2015.06.009 26341566

[51] Monteith LL, Bahraini NH, Gerber HR, Dorsey Holliman B, Schneider AL, Holliday R, et al. Military sexual trauma survivors’ perceptions of Veterans Health Administration care: A qualitative examination. Psychol Serv. 2020 May;17(2):178–86. doi: 10.1037/ser0000290 30265071

[52] Schooley BL, Horan TA, Lee PW, West PA. Rural veteran access to healthcare services: investigating the role of information and communication technologies in overcoming spatial barriers. Perspect Health Inf Manag. 2010 Apr 1;7(Spring):1f. 20697468 PMC2889372

[53] Hussey PS, Ringel JS, Ahluwalia S, Price RA, Buttorff C, Concannon TW, et al. Resources and Capabilities of the Department of Veterans Affairs to Provide Timely and Accessible Care to Veterans. Rand Health Q. 2016 May 9;5(4):14. 28083424 10.7249/rhq-05-04-14 PMC5158229

[54] US Department of Veteran Affairs. About rural Veterans. [Internet]. Office of Rural Health. [cited 2023 May 22]. Available from: https://www.ruralhealth.va.gov/aboutus/ruralvets.asp

[55] Moreau JL, Cordasco KM, Young AS, Oishi SM, Rose DE, Canelo I, et al. The use of telemental health to meet the mental health needs of women using Department of Veterans Affairs services. Womens Health Issues. 2018;28(2):181–7. doi: 10.1016/j.whi.2017.12.005 29339013

[56] U.S. Department of Veterans Affairs. Bridging the Digital Divide. [Internet]. VA Telehealth. [cited 2023 May 23]. Available from: https://telehealth.va.gov/digital-divide

