# Supplementary material for: Perspectives of VA healthcare from rural women veterans not enrolled in or using VA healthcare
Source: PLoS One. 2023 Aug 14;18(8):e0289885. doi: 10.1371/journal.pone.0289885 (PMC10424869; doi:10.1371/journal.pone.0289885)
Supplement: S1 File — (PDF) [file pone.0289885.s001.pdf]

Women's Health Transition Training (WHTT): Women's Health Focus Groups
Focus Group Guide V5: 6/8/2021

Welcome

Hello everyone! Welcome, and thank you for being part of this discussion today.

Introductions

Let's start by introducing our team: I'm ____________ and I will be facilitating the discussion today. Also with us is ________________, who will also be joining the discussion and ___________ who will be taking notes and helping with any technical issues. We are researchers with the VA, and are part of a team that is seeking to better understand how and why women Veterans, particularly those in rural communities, make their choices about where to receive health care. So, as part of that effort we are conducting a series of focus groups like this one. As women Veterans yourself, YOU are the experts here, so we are very interested to hear what you have to say.

Ground Rules

Before we begin, let me mention a few things about how we usually conduct these groups:

1) As I mentioned before, I will be the facilitator for the group. My role is to be neutral in our discussion, to ask the questions we have for the group, and to encourage everyone to participate. I won't be doing much talking, but may ask you to explain more or to give an example. Also, it's my job to see that everyone has a chance to voice their opinions, as well as to keep us moving along so that we have time to discuss all of the questions. So if it ever seems as though I am cutting you off, I want you to know now that this is not meant to be rude at all—I'm just trying to make sure that we have time to have a complete discussion of each question.

2) It's really important that everyone hear this: THERE ARE NO RIGHT OR WRONG ANSWERS!!! Each person's opinions are valid, and we want to hear a wide range of opinions about the questions I'll be asking. So, please speak up, whether you agree or disagree with what's being said, and let us know what you think. There is no penalty for expressing something negative! We view everything you say as constructive feedback.

3) Let me tell you about our recording process. We will be recording our discussion today via our Zoom platform. We record these focus groups because we want to capture everything that all of you say and, despite taking notes, we simply can't write fast enough to get it all down. The audio recordings will be transcribed by a third party, and then all the focus group data will be analyzed together for themes.

4) <u>Throughout the entire process, we will protect your privacy and confidentiality.</u> We'll be using only first names in our discussion today, if we use names at all. And when we put together the results across all groups, we will exclude any names entirely. Also, any identifiable information inadvertently captured during the discussion will be removed during the transcription process.

   <u>So, now that you know what our process is, is everyone OK with being recorded?</u> *(Wait for affirmation. If anyone is not willing to be recorded, they may be excused from the group.)*

5) Great. We plan to be finished with our discussion by **[time]**. I want to be very respectful of your time, so I'll be sure to wrap up by then.

Questions

Okay, now does anyone have any questions about anything I've just said? *(Wait for responses.)*

Are you ready to get started? *(Wait for affirmation.)* Okay, great. I'll start the recording now. After I start recording, I will say aloud for the record that we are recording.

(Start recording.)

Okay, thank you all for being here today to talk about your healthcare preferences and experiences, and thank you for giving your consent for this discussion to be recorded. Can you please confirm that you are aware that this conversation is being recorded and that you have given your verbal consent to participate in this focus group?

Great. Now to get us started, I'd like to know a little more about you:

1. What is your relationship to the military?
    - What branch of the military are/were you in?
    - How long did you serve in the military? When did you separate?

2. Do you identify as a Veteran now? Why or why not?

3. Where do you live now?

4. How did you hear about this focus group opportunity?

Great- thank you. Now I'd like to ask you some questions about how you currently access health care.

5. Since you separated from the military, have you had a need for health care services? For what type(s) of services? Please briefly describe.

6. If/when you need healthcare services, how do you go about determining where to obtain those services?

7. Where do you receive healthcare services when needed? Why have you chosen to receive health care at [location]?

8. How do you currently access healthcare services? How do you find that process?

9. How satisfied are you with the healthcare you currently receive? What do you like and dislike about that care? Why?

10. Besides accessing health care services at _______ , do you engage in any other forms of health care? For instance, do you see alternative medicine providers like massage therapists or acupuncturists or practitioners of homeopathic medicine? (Probe: Tell me about- what kinds of care do you receive there and why?)

Thank you- this is really helpful. Now I'm going to ask you some questions about your opinions about and experiences with VA health care specifically.

11. What comes to mind when you think of the VA as a healthcare entity?

12. What do you know about VA healthcare for women Veterans? What are your opinions of VA healthcare for women Veterans? [Probe how they formed these impressions / obtained such knowledge]

13. Have you ever considered enrolling in VA healthcare? Why or why not?

14. Have you ever attempted to enroll in VA healthcare? Why or why not? If so, how did it go?

15. Would you like to be enrolled in VA healthcare? What would change that for you (i.e., make you want to be enrolled or not want to be enrolled)?

16. For what reasons do you not use the VA for your healthcare?
    - (If needed) In your opinion, why don't some women Veterans in rural areas use the VA for healthcare services?
    - What barriers or challenges to using VA healthcare do rural women Veterans such as yourself experience?
    - What would help rural women Veterans such as yourself to be able to use VA healthcare?

17. What healthcare services would you want to receive at the VA? What types of healthcare services do you think are most important for the VA to provide to rural women Veterans? Why?
    - (If needed) Probe for:
        1. General Medical Care
        2. Preventive Care
            - Such as Mammograms, Pap Smears, Other Cancer Screenings, Cardiovascular Prevention, Immunizations
        3. Reproductive Health Care
            - Contraception
            - Prenatal and maternity care
            - Menopause treatment
        4. Pharmacy
        5. Complementary and Integrative Therapies

6. Mental health
7. Pain Management

18. To what extent would your desire to receive VA healthcare services differ based on whether such services were provided in a mixed-gender settings vs. a women's specific setting?
    - Would this differ based on the type of healthcare service (e.g., primary care vs mental health)?

19. Would your desire to receive VA healthcare services vary based on whether the healthcare provider was specifically trained in women's health issues?

Thank you for sharing this; it is really helpful. We are almost done now. My last few questions focus on your thoughts about how to best communicate with rural women Veterans about VA healthcare.

20. How have you sought out information within your community about Veterans' benefits, healthcare, or general Veterans' issues? (Probe: what/who is your community? Who do you connect with? Is your community Veteran-related or not?)
    - (If needed) What individuals or organizations within your community have you turned to for this type of information?

21. What information or education have you received about the healthcare that the VA offers? [Probe for types, how to access, etc.]

22. How have you received that information? [Probe for method of delivery, source, timing, clarity]
    - Did you find this way of receiving information to be helpful, or would you prefer for it to have been different? How so?

23. Going forward, how would you prefer to learn about VA healthcare services?

24. In your opinion, what would be the best way(s) to inform rural women Veterans in your community about VA healthcare services (e.g., how to enroll, what services the VA providers to women Veterans)? Why?

25. What information do you wish you had had about the VA when deciding whether or not to enroll in VA healthcare?

26. What information do you want to know now about VA healthcare?

27. If you wanted to know more about VA healthcare, where would you go for information? How would you prefer to access this information?

Thank you so much for participating in this discussion today. We really appreciate you sharing your thoughts and experiences. This information is very helpful for us to understand how to improve VA

healthcare services for rural women Veterans. We have covered a lot of ground in our discussion. Before we officially end, is there anything else that you think is important for us to know that we haven't talked about?

Thank you again for your time and insights!
